# Common social determinants for overweight and obesity, and dental caries among adolescents in Northern Norway: a cross-sectional study from the Tromsø Study Fit Futures cohort

**DOI:** 10.1186/s12903-021-01406-5

**Published:** 2021-02-05

**Authors:** Lina Stangvaltaite-Mouhat, Anne-Sofie Furberg, Sergei N. Drachev, Tordis A. Trovik

**Affiliations:** 1 Department of Clinical Dentistry, Faculty of Health Sciences, UiT The Arctic University of Norway, 9037 Tromsø, Norway; 2 Oral Health Centre of Expertise in Eastern Norway, Sørkedalsveien 10A, 0369 Oslo, Norway; 3 Department of Microbiology and Infection Control, University Hospital of North Norway, 9038 Tromsø, Norway; 4 Faculty of Health and Social Sciences, Molde University College, 6410 Molde, Norway; 5 Department of Community Medicine, Faculty of Health Sciences, UiT The Arctic University of Norway, 9037 Tromsø, Norway; 6 Department of Prosthodontics, Northern State Medical University, 163000 Arkhangelsk, Russia

**Keywords:** General health condition, Body mass index, Waist circumference, Oral health condition, Untreated dental caries, Social determinants, Common risk factors, Adolescents

## Abstract

**Background:**

Non-communicable general and oral health conditions share common risk factors. Studies investigating common social determinants as risk factors for overweight/obesity and dental caries among the same adolescents are scarce and inconclusive.

**Methods:**

This cross-sectional study included data from 464 girls and 494 boys from the population-based Tromsø study Fit Futures, which included first-year students attending upper secondary school in 2010–2011 from two municipalities in Northern Norway (1038 participants in total, 93% participation rate). Multivariable binary logistic regression analyses stratified by sex were used to investigate the association between socioeconomic position indicators (adolescent’s own study program, parents’ education and employment status) and overweight/obesity indicated by body weight and waist circumference, untreated dental caries in dentine, and when these conditions were considered simultaneously.

**Results:**

Boys enrolled in the general studies and sports programs (versus vocational) had lower odds of being overweight/obese (POR 0.42, 95% CI 0.20–0.86 and POR 0.24, 95% CI 0.08–0.73, respectively), of having high waist circumference (POR 0.39, 95% CI 0.21–0.75 and POR 0.25, 95% CI 0.10–0.64, respectively), dental caries (POR 0.57, 95% CI 0.32–0.99 and POR 0.47, 95% CI 0.22–0.98, respectively), and being simultaneously overweight/obese, having high waist circumference and dental caries (POR 0.24, 95% CI 0.07–0.81 and POR 0.11, 95% CI 0.01–0.98, respectively). Girls enrolled in the general studies program (versus vocational) had lower odds of having dental caries (POR 0.50, 95% CI 0.30–0.84).

**Conclusions:**

Adolescent’s own study program was identified to be a common social determinant for overweight/obesity and dental caries among boys. These results support the broader concept of social determinants as common risk factors for general and oral health conditions, and call for common health promotion strategies addressing these common social determinants among adolescents. However, there is a need for more studies to investigate and better understand the influence of social determinants on health conditions among adolescents.

## Background

The non-communicable general health conditions overweight and obesity are considered global epidemics [1], and their prevalence among children and adolescents is increasing substantially [2]. In addition to amplifying multiple risk factors (e.g., metabolic syndrome, such as high blood pressure or deteriorated glucose tolerance) in childhood and adolescence, overweight and obesity are risk factors for cardiovascular diseases in adulthood, which are the leading annual cause of death worldwide, according to data from the Global Burden of Diseases Study [3–5]. Dental caries is a non-communicable oral health condition; untreated dental caries in the permanent dentition was reported as the most prevalent medical condition in the Global Burden of Diseases Study in 2015 [6]. Nevertheless, overweight/obesity and dental caries are highly preventable health conditions, and therefore identification of common risk factors for overweight/obesity and dental caries among children and adolescents and addressing them may contribute towards common health promotion strategies.

It has been demonstrated that overweight/obesity and dental caries share common risk factors, such as behavioral variables (e.g., sugar-sweetened beverages are associated with both, overweight/obesity and dental caries) and psychosocial factors (e.g., stress and perceived control) [7]. However, behavioral and psychosocial factors are only intermediary determinants of health, as these factors are shaped by one’s social environment, i.e. the social determinants of health [8–10]. Therefore, social determinants may be interpreted as superior common risk factors for overweight/obesity and dental caries compared to intermediary determinants. According to the World Health Organization (WHO), the social determinants of health are the conditions in which people are born, grow, live, work and age; indicators of socioeconomic position (SEP) lie under the umbrella of social determinants [10]. The findings of studies, which use data from the same study population of children or adolescents to investigate associations between social determinants (including SEP) and both overweight/obesity and dental caries, are scarce and inconclusive. A Swedish cohort study among 4–12-year-old children demonstrated the association between residence area and dental caries, however, the association between residence area and overweight/obesity was not observed [11]. A cross-sectional study among 12-year-olds in South Pacific found ethnicity to be a common risk factor for oral conditions and obesity, when these conditions were analyzed simultaneously [12]. Dental caries was associated with lower household income and less educated parent, while obesity was associated with less educated fathers among 4–7-year-old children in the USA [13]. A recent cross-sectional study among adolescents aged 18 years in Hong Kong demonstrated positive association between overweight/obesity and parents’ employment, but no association was found between dental caries and different SEP indicators, such as parents’ employment and household income; the authors called to include more potential common risk factors when investigating this association [14].

**Fig. 1:**
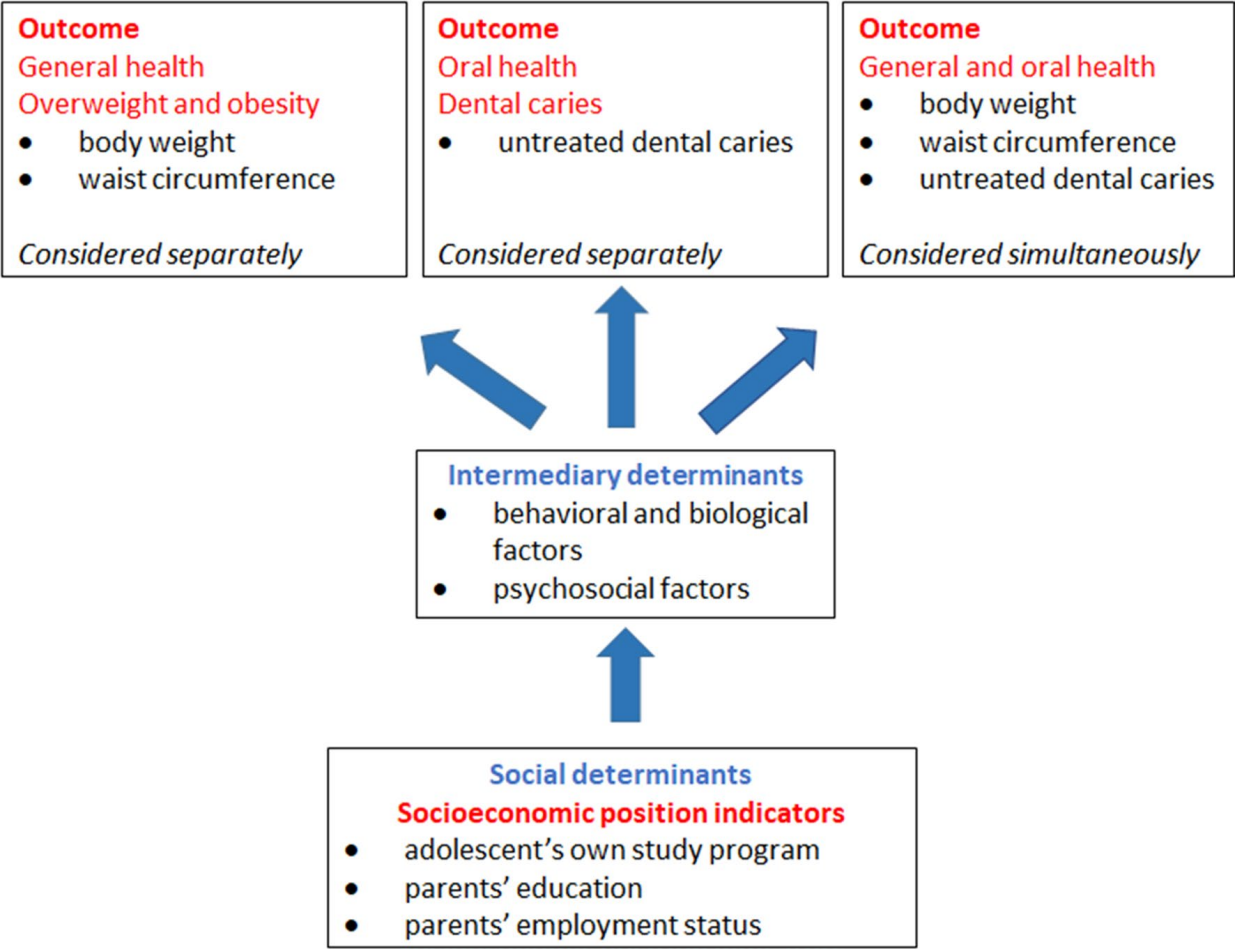
The conceptual framework of the study, based on the WHO conceptual framework on the social determinants of health [10]

To our knowledge, no studies investigated association between social determinants and overweight/obesity, and social determinants and dental caries among the same adolescents using the WHO conceptual model for social determinants of health. Therefore, the aim of the present study was to identify common social determinants for overweight/obesity and dental caries among adolescents in Northern Norway. We investigated the association between SEP indicators (adolescent’s own study program, parents’ education and employment status) and measures of overweight/obesity and abdominal obesity, and untreated dental caries. We employed two approaches: (1) when general and oral health conditions were considered separately, and (2) when general and oral health conditions were considered simultaneously. The conceptual framework of this study, based on the WHO conceptual framework on the social determinants of health [10], is presented in Fig. 1. The hypotheses were: (1) that body weight, waist circumference and untreated dental caries were associated with SEP as measured by the adolescent’s own study program, parents’ education and employment status and the pattern of the association between SEP and overweight/obesity resembled the pattern of the association between SEP and dental caries, when both health conditions were considered separately, and (2) that SEP was associated with overweight/obesity and untreated dental caries when these conditions were considered simultaneously. These hypotheses suggested that overweight/obesity and untreated dental caries share common social determinants as common risk factors.

## Methods

### Study design, and population

This cross-sectional study included data from a population-based cohort study, the Tromsø Study Fit Futures (FF1). FF1 was conducted in the Tromsø school district, Northern Norway (the urban Tromsø municipality and the rural Balsfjord municipality) [15]. All first-year students attending upper secondary school (seven schools in Tromsø and one in Balsfjord) in 2010–2011, mainly aged 15–19 years, were invited to participate in FF1. Of a total of 1117 invited students, 1038 participated (93% participation rate), while 1010 (498 girls and 512 boys) volunteered to participate in the oral health part (90% participation rate), which consisted of a dental evaluation [16, 17]. Data on general health was collected at the Clinical Research Unit, University Hospital of North Norway [16]; data on dental health was collected at the University Dental Clinic [17]. All procedures were done during school hours [17]. After exclusion of participants aged 19 years or older, and those with missing data on height, weight, waist circumference and untreated dental caries, 464 girls and 494 boys (86% participation rate) were eligible for inclusion in the analyses.

### Variables and measurements

#### Socioeconomic position indicators, demographic, behavioral, biological and psychosocial factors

SEP indicators (adolescent’s own study program, parents’ education, and employment status) were taken from the pretested, electronic self-administered FF1 questionnaire, as were demographic characteristics (age, sex, birth country, and household composition) [Furberg 2010, cited in 18] (Additional file 1: Table 1, Additional file 2: Table 2).

Following the WHO conceptual framework for the social determinants of health, as intermediary determinants, twelve behavioral, biological and psychosocial variables were selected: history of chronic disease, alcohol intake, smoking, snuff use, physical activity, sugar-containing sweets and beverages intake, other dietary factors (including intake of omega 3 fatty acids-rich food/supplements, dairy products, fruits/vegetables, vitamin or mineral supplements), tooth brushing frequency, dental satisfaction and self-esteem, psychological therapy and sleep sufficiency, which were taken from the FF1 questionnaire, and vitamin D status, which was measured as serum vitamin D (25-hydroxyvitamin D) level (Additional file 1: Table 1, Additional file 2: Table 2).

#### Outcome variables

Body mass index (BMI) was used to assess body weight categories. Height and weight were measured to the nearest 0.1 cm and 0.1 kg, respectively, on an automatic electronic scale/stadiometer (Jenix DS 102 stadiometer, Dong Sahn Jenix, Seoul, Korea), with participants wearing light clothing and no footwear [19]. BMI was calculated based on the WHO index for students aged 18 years [20]. For 15–17-year-olds, International Obesity Task Force age- and sex-specific cut-off values were used [21]. BMI was categorized as underweight (corresponding to adult BMI < 18.5 kg/m^2^), normal weight (adult BMI value 18.5–24.9 kg/m^2^), overweight (adult BMI value 25–29.9 kg/m^2^), and obese (adult BMI value ≥ 30 kg/m^2^). In binary logistic regression analysis, this variable was dichotomized into *normal weight* (corresponding to adult BMI value < 25 kg/m^2^) and *overweight/obese* (corresponding to adult BMI value ≥ 25 kg/m^2^) [22].

Abdominal obesity was expressed by waist circumference, which was measured to the nearest centimeter with a measuring tape placed horizontally at umbilical level and at the end of a normal expiration. Subjects were standing with arms relaxed at sides and weight evenly distributed across feet [19]. Waist circumference was measured twice, and the mean value was used in the analyses. For 15–18-year-olds, waist circumference was categorized into *normal* and *high* based on age- and sex-specific cut-off values [23].

The oral health outcome, untreated dental caries, was expressed as a decayed teeth (DT) component of a decayed, missed, filled teeth (DMFT) index, which was recorded by a single dentist [17]. Untreated dental caries was detected clinically and radiographically using caries grading system suggested by Amarante and colleagues [24]. Untreated dental caries in enamel was graded level 1–2 referring to ICDAS level 1–3, while untreated dental caries in dentine was graded level 3–5, corresponding to ICDAS level 4–6 [25]. For analysis, D_3–5_ T component was dichotomized into *no untreated dental caries in dentine* (D_3–5_ T = 0) and *untreated dental caries in dentine* (D_3–5_ T > 0).

BMI, abdominal obesity and untreated dental caries were considered simultaneously and combined into one general and oral health outcome variable, which was categorized into *normal weight, normal waist circumference and no untreated dental caries in dentine* and o*verweight/obese, high waist circumference and untreated dental caries in dentine.*

### Statistical analysis

Statistical analyses were performed in Statistical Package for the Social Sciences (SPSS, Version 26.0, IBM Corp., Armonk, NY, USA). Chi-square test was used for categorical variables to analyze the differences in SEP indicators, demographic characteristics, behavioral, biological and psychosocial factors, body weight, abdominal obesity and untreated dental caries between girls and boys. The binary logistic regression analysis was stratified by sex, as it has been shown that health behavior differs between adolescent girls and boys [26, 27]. Univariable binary logistic regression analysis was used to identify the characteristics associated with the outcomes.

Four multivariable binary logistic regression models were constructed to find the associations between body weight, waist circumference, untreated dental caries and combined general and oral health outcome (dependent variables), and SEP indicators (independent variables) adjusted for demographic characteristics (independent of significance), and for all behavioral, biological and psychosocial factors that achieved statistical significance p ≤ 0.2 in the univariable binary logistic regression analyses for that sex. The hierarchical regression (blockwise method) was used. All SEP indicators were entered in the first block and the selected covariates were placed in the second block. The assumption of multicollinearity (tolerance, VIF statistics and eigenvalues) was not violated in any of the models [28]. The Hosmer–Lemeshow goodness-of-fit statistic yielded *p* > 0.05 for all models constructed. Nagelkerke R^2^ was recorded for the first (SEP) block and the whole adjusted model for every outcome [28]. The level of significance was set at *p* = 0.05 and prevalence odds ratios (PORs) are presented with 95% confidence intervals (CIs).

#### Ethical considerations

FF1 was performed in compliance with Good Clinical Practice and the Declaration of Helsinki. The Norwegian Data Protection Authority (reference number 2009/1282) and the Regional Committee of Medical and Health Research Ethics (reference number 2011/1702/REK nord) approved the study at start-up. Participation was based on signed written informed consent: participants aged 16 years and above signed themselves, and younger participants brought written permission from their guardians. The present study was approved by the Regional Committee of Medical and Health Research Ethics (reference number 2018/172/REK nord).

## Results

### Sample characteristics

After exclusion of participants aged 19 years or older, and those with missing data on height, weight, waist circumference and untreated dental caries, 464 girls and 494 boys (86% participation rate) were eligible for inclusion in the analyses. The mean age of included girls was 16.16 (standard deviation (SD) 0.48) years and for the 494 included boys it was 16.11 (SD 0.56) years (Additional file 2: Table 2). There was a difference in adolescent’s own study program between girls and boys, but the proportion of girls and boys was the same in regard to parents’ education and employment. A higher proportion of boys had untreated dental caries in dentine, a higher proportion of girls had high waist circumference and there was a difference in body weight between the sexes (Table 1).

**Table 1:** Socioeconomic position indicators, body weight, waist circumference, and untreated dental caries in dentine among the participants of The Tromsø Study Fit Futures 1 (FF1) stratified by sex

Characteristics	Girls	Boys
	N (%)	N (%)
Socioeconomic position indicators		
Adolescent’s own study program*	464	494
General studies	238 (51)	150 (31)
Sports	38 (8)	66 (13)
Vocational	188 (41)	278 (56)
Mother’s education	458	482
Do not know	103 (23)	140 (29)
High school or less	156 (34)	157 (33)
College less than 4 years	88 (19)	81 (17)
College 4 years or more	111 (24)	104 (21)
Father’s education	450	475
Do not know	129 (29)	139 (29)
High school or less	173 (38)	187 (40)
College less than 4 years	60 (13)	63 (13)
College 4 years or more	88 (20)	86 (18)
Parents’ employment	415	440
Both parents work full time	252 (61)	266 (60)
At least one parent does not work full time	163 (39)	174 (40)
General health outcome		
Body weight (BMI)*	464	494
Underweight	10 (2)	40 (8)
Normal weight	343 (74)	341 (69)
Overweight	78 (17)	76 (15)
Obese	33 (7)	37 (8)
Waist circumference*	464	494
Normal	298 (64)	372 (75)
High	166 (36)	122 (25)
Oral health outcome		
Untreated dental caries in dentine (D_3-5_ T) *	464	494
No (D_3-5_ T = 0)	296 (64)	273 (55)
Yes (D_3-5_ T > 0)	168 (36)	221 (45)
Combined general and oral health outcome	245	268
Normal weight/normal waist circumference/no untreated dental caries in dentine	192 (78)	211 (79)
Overweight/obese/high waist circumference/untreated dental caries in dentine	53 (22)	57 (21)

*p < 0.05 according to Chi-square test between girls and boys in FF1

### Body weight, waist circumference and untreated dental caries when conditions were considered separately

**Fig. 2:**
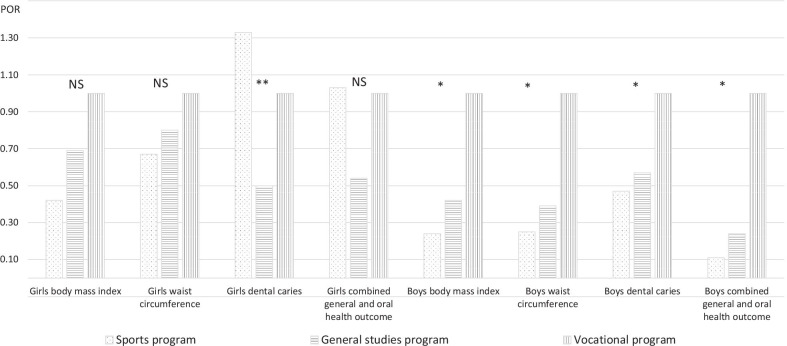
Associations between body weight, waist circumference, untreated dental caries and combined general and oral health outcome among girls and boys based on the socioeconomic position (SEP) indicator (adolescent’s own study program) in the Tromsø Study Fit Futures 1. The prevalence odds ratios derived from the multivariable binary logistic regression analyses. *p < 0.05 general studies program versus vocational program, and sports program versus vocational program. **p < 0.05 general studies program versus vocational program. *NS* not statistically significant

Boys enrolled in the general studies program (versus vocation program) had 58% lower odds of being overweight/obese (POR 0.42, 95% CI 0.20–0.86), 61% lower odds of having high waist circumference (POR 0.39, 95% CI 0.21–0.75), and 43% lower odds of having untreated dental caries (POR 0.57, 95% CI 0.32–0.99) (Tables 2, 3, 4, Fig. 2). Being enrolled in the sports program (versus vocational program) was negatively associated with being overweight/obese (POR 0.24, 95% CI 0.08–0.73), having high waist circumference (POR 0.25, 95% CI 0.10–0.64), and untreated dental caries (OR 0.47, 95% CI 0.22–0.99) (Tables 2, 3, 4, Fig. 2).

**Table 2:** Crude and adjusted associations between age- and sex-adjusted body weight (body mass index, BMI) and socioeconomic position (SEP) indicators in the study sample, stratified by sex

Characteristic	Girls		Boys	
	Crude POR (95% CI)	Adjusted^a^ POR (95% CI) N = 341	Crude POR (95% CI)	Adjusted^b^ POR (95% CI) N = 356
Socioeconomic position indicators				
Adolescent’s own study program				
Vocational	1	1	1	1
General studies	0.66 (0.42–1.03)	0.69 (0.37–1.31)	0.41 (0.25–0.69)	0.42 (0.20–0.86)
Sports	0.37 (0.14–0.99)	0.42 (0.11–1.56)	0.19 (0.07–0.48)	0.24 (0.08–0.73)
Mother’s education				
College 4 year or more	1	1	1	1
College less than 4 years	0.92 (0.47–1.77)	0.60 (0.24–1.48)	2.35 (1.07–5.18)	3.49 (1.29–9.49)
High school or less	1.04 (0.59–1.83)	0.54 (0.23–1.28)	2.71 (1.35–5.45)	2.18 (0.87–5.50)
Do not know	0.95 (0.50–1.77)	0.39 (0.13–1.16)	2.86 (1.41–5.79)	1.24 (0.40–3.82)
Father’s education				
College 4 year or more	1	1	1	1
College less than 4 years	1.77 (0.80–3.93)	2.35 (0.81–6.76)	0.86 (0.35–2.13)	0.45 (0.14–1.38)
High school or less	1.56 (0.81–3.01)	2.04 (0.78–5.34)	1.68 (0.87–3.25)	0.89 (0.37–2.13)
Do not know	1.74 (0.88–3.44)	3.03 (0.96–9.53)	2.08 (1.05–4.10)	1.57 (0.49–5.00)
Parents’ employment				
Both full time	1	1	1	1
At least 1 not full time	1.25 (0.79–1.98)	1.07 (0.60–1.93)	1.57 (1.00–2.46)	0.96 (0.55–1.71)
Nagelkerke R^2^ first (SEP) block	0.056		0.118	
Nagelkerke R^2^ whole model	0.117		0.164	

0_normal weight (corresponding to adult BMI value < 25 kg/m^2^), 1_overweight/obese (corresponding to adult BMI value ≥ 25 kg/m^2^). Crude prevalence odds ratios are presented according to univariable and adjusted prevalence odds ratios according to multivariable binary logistic regression analyses. The number of participants in each analysis differs due to missing data

^a^Adjusted by all SEP indicators, age, demographic characteristics (independent of significance), and behavioral, biological and psychosocial factors that proved statistical significance *p* ≤ 0.2 in univariable binary logistic regression analysis (tooth brushing frequency, psychological therapy, vitamin D)

^b^Adjusted by all SEP indicators, age, demographic characteristics (independent of significance), behavioral, biological and psychosocial factors that proved statistical significance *p* ≤ 0.2 in univariable binary logistic regression analysis (alcohol intake, snuff use, sugar-containing sweets and beverages, other dietary factors, tooth brushing frequency, vitamin D)

**Table 3:** Crude and adjusted associations between age- and sex-adjusted waist circumference and socioeconomic position (SEP) indicators stratified by sex

Characteristic	Girls		Boys	
	Crude POR (95% CI)	Adjusted^a^ POR (95% CI) N = 380	Crude POR (95% CI)	Adjusted^b^ POR (95% CI) N = 384
Socioeconomic position indicators				
Adolescent’s own study program				
Vocational	1	1	1	1
General studies	0.65 (0.44–0.96)	0.80 (0.46–1.41)	0.40 (0.24–0.67)	0.39 (0.21–0.75)
Sports	0.56 (0.26–1.20)	0.67 (0.26–1.74)	0.36 (0.16–0.71)	0.25 (0.10–0.64)
Mother’s education				
College 4 year or more	1	1	1	1
College less than 4 years	0.96 (0.53–1.72)	0.97 (0.44–2.12)	1.67 (0.82–3.40)	2.22 (0.95–5.18)
High school or less	1.19 (0.72–1.97)	1.25 (0.60–2.60)	1.63 (0.88–3.04)	1.31 (0.61–2.82)
Do not know	0.91 (0.52–1.60)	1.04 (0.41–2.67)	1.91 (1.02–3.58)	0.98 (0.38–2.57)
Father’s education				
College 4 year or more	1	1	1	1
College less than 4 years	1.31 (0.65–2.16)	1.54 (0.65–3.66)	1.03 (0.45–2.36)	0.69 (0.25–1.86)
High school or less	1.43 (0.83–2.47)	1.18 (0.55–2.53)	1.47 (0.78–2.77)	0.96 (0.43–2.16)
Do not know	1.21 (0.68–2.16)	1.31 (0.51–3.36)	1.96 (1.02–3.76)	1.28 (0.44–3.72)
Parents’ employment				
Both full time	1	1	1	1
At least 1 not full time	1.15 (0.77–1.74)	1.05 (0.64–1.74)	1.49 (0.96–2.30)	1.13 (0.66–1.93)
Nagelkerke R^2^ first (SEP) block	0.018		0.089	
Nagelkerke R^2^ whole model	0.044		0.089	

0_waist circumference normal, 1_waist circumference high; based on age- and sex-specific cut-off values. Crude prevalence odds ratios are presented according to univariable and adjusted prevalence odds ratios according to multivariable binary logistic regression analyses. The number of participants in each analysis differs due to missing data

^a^Adjusted by all SEP indicators, age, demographic characteristics (independent of significance), and behavioral, biological and psychosocial factors that proved statistical significance *p* ≤ 0.2 in univariable binary logistic regression analysis (snuff use, tooth brushing frequency, dental satisfaction and self-esteem)

^b^Adjusted by all SEP indicators, age, demographic characteristics (independent of significance), behavioral, biological and psychosocial factors that proved statistical significance *p* ≤ 0.2 in univariable binary logistic regression analysis (alcohol use, snuff use, physical activity, other dietary factors, tooth brushing frequency)

**Table 4:** Crude and adjusted associations between untreated dental caries in dentine (D_3-5_ T) and socioeconomic position (SEP) indicators stratified by sex

Characteristic	Girls		Boys	
	Crude POR (95% CI)	Adjusted^a^ POR (95% CI) N = 364	Crude POR (95% CI)	Adjusted^b^ POR (95% CI) N = 371
Socioeconomic position indicators				
Adolescent’s own study program				
Vocational	1	1	1	1
General studies	0.41 (0.27–0.61)	0.50 (0.30–0.84)	0.53 (0.35–0.79)	0.57 (0.32–0.99)
Sports	0.92 (0.46–1.85)	1.33 (0.59–3.00)	0.50 (0.29–0.87)	0.47 (0.22–0.98)
Mother’s education				
College 4 year or more	1	1	1	1
College less than 4 years	1.02 (0.56–1.86)	1.15 (0.54–2.47)	1.18 (0.65–2.15)	1.65 (0.75–3.65)
High school or less	1.57 (0.94–2.61)	1.43 (0.70–2.92)	1.74 (1.05–2.90)	1.93 (0.95–3.92)
Do not know	1.03 (0.58–1.82)	1.07 (0.43–2.66)	1.76 (1.05–2.96)	1.48 (0.60–3.61)
Father’s education				
College 4 year or more	1	1	1	1
College less than 4 years	1.39 (0.67–2.88)	1.18 (0.48–2.86)	0.73 (0.37–1.41)	0.46 (0.20–0.90)
High school or less	2.14 (1.21–3.78)	1.74 (0.81–3.72)	1.10 (0.66–1.84)	0.57 (0.29–1.13)
Do not know	1.78 (0.98–3.24)	1.08 (0.41–2.88)	1.14 (0.67–1.96)	0.71 (0.29–1.74)
Parents’ employment				
Both full time	1	1	1	1
At least 1 not full time	1.01 (0.67–1.53)	0.85 (0.51–1.42)	1.66 (1.13–2.44)	1.49 (0.94–2.36)
Nagelkerke R^2^ first (SEP) block	0.087		0.080	
Nagelkerke R^2^ whole model	0.120		0.134	

0_waist circumference normal, 1_waist circumference high; based on age- and sex-specific cut-off values. Crude prevalence odds ratios are presented according to univariable and adjusted prevalence odds ratios according to multivariable binary logistic regression analyses. The number of participants in each analysis differs due to missing data

^a^Adjusted by all SEP indicators, age, demographic characteristics (independent of significance), and behavioral, biological and psychosocial factors that proved statistical significance *p* ≤ 0.2 in univariable binary logistic regression analysis (smoking, snuff use, sugar-containing sweets and beverages, other dietary factors, tooth brushing frequency, psychological therapy)

^b^Adjusted by all SEP indicators, age, demographic characteristics (independent of significance), behavioral, biological and psychosocial factors that proved statistical significance *p* ≤ 0.2 in univariable binary logistic regression analysis (alcohol intake, smoking, snuff use, physical activity, sugar-containing sweets and beverages, other dietary factors, tooth brushing frequency, dental satisfaction and self-esteem, psychological therapy, sleep sufficiency)

Boys who had mother with a lower education level (college less than 4 years versus college 4 years or more) had more than three times higher odds of being overweight/obese (POR 3.49, 95% CI 1.29–9.49) (Table 2) and boys who had father with lower education level (college less than 4 years vs college 4 years or more) had 54% lower odds to have untreated dental caries (POR 0.46, 95% CI 0.20–0.90) (Table 4).

Girls enrolled in the general studies program versus the vocational program had 50% lower odds of having untreated dental caries (POR 0.50, 95% CI 0.30–0.84) (Table 4, Fig. 2).

### Body weight, waist circumference and untreated dental caries when conditions were considered simultaneously

Boys enrolled in the general studies program (versus vocation program) had 76% lower odds (POR 0.24, 95% CI 0.07–0.81) and boys enrolled in the sports program (versus vocational program) had 89% lower odds (POR 0.11, 95% CI 0.01–0.98) of being simultaneously overweight/obese, having high waist circumference and untreated dental caries in dentine (Table 5). Boys who had mother with a lower education level (college less than 4 years versus college 4 years or more) had seven times higher odds of being simultaneously overweight/obese, having high waist circumference and untreated dental caries in dentine (POR 7.13, 95% CI 1.34–37.90) (Table 5).

**Table 5:** Crude and adjusted associations between combined general and oral health outcome and socioeconomic position (SEP) indicators stratified by sex

Characteristic	Girls		Boys	
	Crude POR (95% CI)	Adjusted^a^ POR (95% CI) N = 172	Crude POR (95% CI)	Adjusted^b^ POR (95% CI) N = 196
Socioeconomic position indicators				
Adolescent’s own study program				
Vocational	1	1	1	1
General studies	0.33 (0.17–0.63)	0.54 (0.21–1.38)	0.23 (0.10–0.49)	0.24 (0.07–0.81)
Sports	0.45 (0.12–1.72)	1.03 (0.17–6.31)	0.11 (0.03–0.47)	0.11 (0.01–0.98)
Mother’s education				
College 4 year or more	1	1	1	1
College less than 4 years	0.69 (0.24–1.98)	0.69 (0.15–3.14)	3.68 (1.08–12.61)	7.13 (1.34–37.90)
High school or less	1.66 (0.76–3.64)	1.83 (0.52–6.49)	5.28 (1.72–16.21)	4.02 (0.76–21.13)
Do not know	0.92 (0.38–2.24)	0.87 (0.22–3.42)	4.76 (1.50–15.09)	0.42 (0.06–3.10)
Father’s education				
College 4 year or more	1	1	1	1
College less than 4 years	1.95 (0.59–6.47)	3.07 (0.64–14.68)	0.73 (0.37–1.41)	0.18 (0.03–1.22)
High school or less	2.30 (0.89–5.92)	1.90 (0.45–7.93)	1.10 (0.66–1.84)	0.34 (0.08–1.49)
Do not know	2.42 (0.94–6.25)	2.32 (0.47–11.46)	1.14 (0.67–1.96)	4.11 (0.48–35.36)
Parents’ employment				
Both full time	1	1	1	1
At least 1 not full time	1.16 (0.59–2.28)	0.82 (0.35–1.94)	1.75 (0.91–3.37)	0.65 (0.25–1.66)
Nagelkerke R^2^ first (SEP) block	0.102		0.233	
Nagelkerke R^2^ whole model	0.182		0.339	

0_Normal weight/normal waist circumference/no untreated dental caries in dentine , 1_Overweight/obese/high waist circumference/untreated dental caries in dentine. Crude prevalence odds ratios are presented according to univariable and adjusted prevalence odds ratios according to multivariable binary logistic regression analyses. The number of participants in each analysis differs due to missing data

^a^Adjusted by all SEP indicators, age, demographic characteristics (independent of significance), behavioral, biological and psychosocial factors that proved statistical significance *p* ≤ 0.2 in univariable binary logistic regression analysis (snuff use, physical activity, other dietary factors, tooth brushing frequency, psychological therapy, sleep sufficiency)

^b^Adjusted by all SEP indicators, age, demographic characteristics (independent of significance), behavioral, biological and psychosocial factors that proved statistical significance *p* ≤ 0.2 in univariable binary logistic regression analysis (alcohol intake, smoking, snuff use, physical activity, other dietary factors, tooth brushing frequency, vitamin D)

## Discussion

The present study, conducted among first-year students attending upper secondary school in Northern Norway, identified adolescent’s own study program as a common social determinant for overweight/obesity and untreated dental caries among boys. Mother’s education was identified as a common social determinant also among boys and only when general and oral health conditions were considered simultaneously.

### Methodological considerations

To our knowledge, this is the first study investigating association between SEP indicators and overweight and obesity, and untreated dental caries among the same adolescents using the WHO conceptual model for social determinants of health employing two approaches, when general and oral health conditions were analyzed separately and simultaneously. The two approaches gave similar results, suggesting that both strategies may be used to investigate common social determinants of general and oral health, however using the model where general and oral health outcomes were considered simultaneously resulted in higher Nagelkerke R^2^.

All general and oral health outcomes were measured using objective criteria during clinical examination. Moreover, to detect untreated dental caries in dentine, radiographies were used.

However, the study has some limitations. This was a cross-sectional study, whose design in general is prone to confounding and does not allow to establish causality [29]. To control for confounders, we used a multivariable binary logistic analysis [29]. Given a high prevalence of our outcomes (more than 10%), results of the current study are interpreted in terms of prevalence odds ratios.

The initial participation rate in FF1 was high, reaching 93%, and participation in the oral health part of FF1 was only slightly lower (90%). It is possible that this decrease in attendance to the dental evaluation that constituted the oral health part of FF1 was associated with low parental education, unemployment, and low income [30]. After exclusions, our final study sample represented 86% of all students invited to FF1, but in multivariable binary logistic regression, the number of participants was reduced to 63% among girls and 62% among boys due to missing data; therefore self-selection bias cannot be ruled out. The sample was collected from both a densely populated urban area (Tromsø, seven schools) and a sparsely populated rural area (Balsfjord, one school) including all upper secondary schools in Tromsø school district, Troms County, Northern Norway. In this county, 29% of the population resides in sparsely populated areas; therefore, the population residing in densely populated areas might be overrepresented in the study sample (7 schools in urban area versus 1 school in rural area). It has been shown that living in densely populated areas is associated with higher physical activity and thus probably better health outcomes among adolescents in Norway [26]. It must be noted that 16–18% of adolescents in Troms County do not live in their parents’ household. Indeed, as Troms County is large, adolescents sometimes have to move from where their parents live to where the school is located – creating the household composition of “living without adults”. This living situation occurs due to adolescents’ need for education; not necessarily because they have a higher level of maturity and hence, their health may be jeopardized. It has been also shown that having an immigrant background was related to worse general and oral health outcomes among children and adults in Norway [31–33]. In Tromsø municipality in 2012, 4.8% of immigrants were aged 16–19 years [34]. In our study sample 6% of girls and 5% of boys reported that they were born outside Norway, indicating that our sample might be representative of the national population with respect to immigrant background.

A pretested, electronic, self-administered questionnaire was employed to collect data on SEP indicators and most of the covariates. Structure and content of the questionnaire were to a large degree adapted from the Tromsø Study among adults [35]. In general, questionnaires are prone to bias, especially regarding sensitive data, like alcohol intake and tobacco use. However, self-administration has been shown to decrease reporting bias [36].

Previous Norwegian study investigated association between health behavior and SEP among adolescents and suggested that adolescent’s own study program in upper secondary school is a potential proxy of an adolescent’s SEP [37]. Therefore, study program was chosen as the SEP indicator in this study. In the Norwegian school system, there is a lawful right, but not an obligation, to complete 1 year of upper secondary school. Students can apply for a general studies program, including a sub-path of a sports, or a vocational study program. The general studies program gives possibility for admission to higher education after three years. At the vocational study program, normally after two years of school training, a student goes in apprenticeship for two years. The completion rates (by normative length of study) differ according to study path (75% in general study program, 37% in vocational study program, during 2013–2018, respectively), and varies by sex, geographic area and parent’s education. Adolescents’ choice of study program has been shown to correlate with their social background [38] and health-related behaviors [37, 39]. As in the present study, the same previous Norwegian study also indicated that parents’ education and occupation are applicable when investigating association between SEP and health outcomes among adolescents [44]. In addition, in Norway, previous studies also have shown that mother’s and father’s education associated with child’s health behavior and adverse health events [37, 40, 41]. We had no data on parents’ occupation, therefore, parents’ employment was used as a substitute variable in this study, as it has been associated with health and health behaviors among adolescents [42].

In this study, one of the indicators of the general health condition, overweight and obesity, body weight (expressed by BMI), was measured. BMI is commonly used as an indicator of overweight and obesity. Indeed, BMI is a ratio between weight and height, and it cannot distinguish between body fatness and fat-free mass [43]. On the other hand, it has been shown that BMI-for-age was a good indicator of body fatness, especially among heavier children and adolescents [44]. In addition to BMI, we used waist circumference as another general health indicator. Waist circumference is a specific measure to define abdominal fatness [45]. In our study, the two measures gave quite similar results, but using BMI presented results with a higher Nagelkerke R^2^ implying that the variability of the studied independent variables explained to a greater extent the variability in BMI than in waist circumference.

In this study, the indicator of the oral health condition, dental caries, was untreated caries in dentine (D_3–5_ T); it was measured and expressed as DT component of DMFT index. The DT component reflects the treatment need, or in other words, the severity of disease, but does not take into consideration dental caries experience (filled and missing due to caries teeth).

### Discussion of the results

Among boys, the statistically significant associations were observed between study program and all the outcomes, *i.e.* body weight, waist circumference, untreated dental caries, and combined general and oral health outcome. It must be noted, that adolescents enrolled in the sports program may have a better general health because of the fact that they are in this study program. Previous prospective Norwegian cohort study demonstrated association between admission to given study programs and health behaviors [37], and our study showed association between study program, and general and oral health outcomes among boys. This finding may be explained that the choice of study program has been shown to depend mainly on the occupation of role models, role models for adolescents being mostly their friends and acquaintances, persons from the same social environment [46]. Therefore, one may assume that not the study program itself is a risk factor of poor general and oral health, but the social context that leads the adolescent to choose a particular program.

Lower mother’s education, another SEP indicator used in this study, was associated with higher BMI and combined general and oral health outcome also only among boys. This finding might refer to gender orientation in adolescents’ behavior. It might be that boys are less mature and more dependent on their mothers, as mothers have been shown to be “the prime mover in the health and welfare of the child” [47]. Our findings regarding mother’s education and boy’s health is in contrast to a study from the USA, in which father’s health-risk lifestyle, which consisted of diet, physical activity, smoking, alcohol use, and sleep, affected boys’ health-risk behavior, while mother’s behavior affected girls’ behavior; however parents’ health-risk lifestyle was not included in this study [48]. It has been shown in Norway that father’s occupation predicted changes in health behavior among 13–21-year-old girls [37]. We may speculate that father’s occupation is linked to father’s education, in the present study, higher father’s education was associated with untreated dental caries among boys, and this finding is in contrast with the previously mentioned study. Even though it has been demonstrated that only few adolescents based their choice of the study program on their parents’ opinions, the indirect influence may not be ruled out [46]. The associations between parents’ education, and general and oral health conditions should be interpreted with caution given a high proportion of the adolescents who did not know or did not report the education level of their parents. Given the differences across genders of parents and children, future studies investigating association between SEP and health outcomes in adolescents should address the issue of gender in the relation between parents and their children.

A study in Hungary showed that incomplete parental employment (unemployed, retired, housewife) resulted in inconsistent associations; it was positively associated with health conditions, like depressive and psychosomatic symptoms, but negatively associated with behavioral factors, like smoking, drinking, and drug use among adolescents [42]. Contrary, a study among adolescents in Hong Kong found the association between full time employed parents and overweight/obesity, when both sexes were analyzed together [14]. In the present study parents’ employment, another SEP indicator, did not associate with any of the outcomes.

A recent study among chief dental officers showed that the majority of the countries acknowledged common risk factor approach when implementing shared preventive strategies for general and oral health, however the approach was interpreted too narrow as strategies addressed mainly intermediary rather than social determinants of health [49]. Our results suggest that public health policymakers should focus on common health promotion strategies for general and oral health that would address common social determinants for general and oral health conditions.

## Conclusions

Adolescent’s own study program was identified to be a common social determinant for overweight/obesity and dental caries among boys. These results support the broader concept of social determinants as common risk factors for general and oral health conditions, and call for common health promotion strategies addressing these common social determinants among adolescents. However, there is a need for more studies to investigate and better understand the influence of social determinants on general and oral health conditions among adolescents.

## Supplementary information

**Additional file 1: Table 1**. Operationalization of variables for statistical analyses. The variables were measured or taken from the questionnaire.

**Additional file 2: Table 2**. Demographic characteristics, behavioral, biological and psychosocial factors among the participants of The Tromsø Study Fit Futures 1 (FF1) stratified by sex.

## Data Availability

The Fit Futures datasets used and analyzed during the current study were supplied by “Helsefak ISM Tromsøundersøkelsen” under the agreement and so cannot be made freely available. Requests for access to these data should be made to tromsous@uit.no.

## Abbreviations

BMI: Body mass index
CI: Confidence interval
DMFT: Decayed, missed and filled teeth
DT: Decayed teeth
FF1: Fit Future 1
POR: Prevalence odds ratio
SD: Standard deviation
SEP: Socioeconomic position
WHO: World Health Organization
