# Supplementary material for: Common social determinants for overweight and obesity, and dental caries among adolescents in Northern Norway: a cross-sectional study from the Tromsø Study Fit Futures cohort
Source: BMC Oral Health. 2021 Feb 5;21:53. doi: 10.1186/s12903-021-01406-5 (PMC7866637; doi:10.1186/s12903-021-01406-5)
Supplement: Supplementary file 1 — Additional file 1: Table 1. Operationalization of variables for statistical analyses. The variables were measured or taken from the questionnaire. [file 12903_2021_1406_MOESM1_ESM.docx]

Supplement Table 1. Operationalization of variables for statistical analyses. The variables were measured or taken from the questionnaire.

| Variable | Measurement | Categorization for statistical analysis |
| --- | --- | --- |
| **Socioeconomic position indicators** | | |
| ***Mother’s education*** | *“What is the highest education completed by your mother?*  *1 Primary school 9 years*  *2 Occupational high school*  *3 High school*  *4 College less than 4 years*  *6 College 4 years or more*  *7 Don’t know* | *1. College 4 year or more*  *2.* *College less than 4 years*  *3. High school or less*  *4. Do not know* |
| ***Father’s education*** | *“What is the highest education completed by your mother?*  *1 Primary school 9 years*  *2 Occupational high school*  *3 High school*  *4 College less than 4 years*  *6 College 4 years or more*  *7 Don’t know* | *1. College 4 year or more*  *2. College less than 4 years*  *3. High school or less*  *4. Do not know* |
| ***Parents’ employment*** | *“Does your mother/father work?”*  *1 Full time*  *2 Part time*  *3 Unemployed*  *4 Disabled*  *5 Domestic*  *6 Attend school/courses*  *7. Pensioned*  *8. Deceased*  *9. Don’t know*  *10. Other* | *1 Both parents work full time*  *2 At least one parent does not work full time* |
| ***Study program*** | *“Main high school program”*  *1 Program for specialization in general studies*  *2 Program for sports and physical education*  *3 Vocational program* | *1 General studies*  *2 Sports*  *3 Vocational* |
| **Demographic characteristics** | | |
| ***Age*** | *“How old are you”?* | *Continuous* |
| ***Birth country*** | *“Were you born in Norway?”*  *1 Yes*  *2 No*  *9 I do not know* | *1 Yes*  *2 No* |
| ***Household composition*** | *“Who do you live with now?”*  *1.Mother*  *2. Father*  *3. 1-2 siblings*  *4. 3 or more siblings*  *5. Mather’s new partner*  *6. Father’s new partner*  *7. Foster parents*  *8. Adoptive parents*  *9. Grandparents*  *10. Friends*  *11. Alone*  *12. In an institution*  *13. Other* | *1. With adults*  *2. Without adults* |
| **Behavioral, biological and psychosocial factors** | | |
| ***History of chronic disease*** | *“Do you have any chronic or persistent disease?”*  *1.Yes*  *2.No* | *0. No*  *1. Yes* |
| ***Alcohol intake*** | *“How often do you drink alcohol?”*  *1.Never*  *2. One per month or less*  *3. 2-4 times per month*  *4. 2-3 times per week*  *5. 4 or more times per week* | *0. Never*  *1. Sometimes* |
| ***Smoking*** | *“Do you smoke?”*  *1. No, never*  *2. Sometimes*  *3. Daily* | *0. Never*  *1. Sometimes/daily* |
| ***Snuff use*** | *“Do you use snuff?”*  *1. No, never*  *2. Sometimes*  *3. Daily* | *0. Never*  *1. Sometimes/daily* |
| ***Physical activity according to Gothenburg instrument*** | *“Exercise and physical exertion in leisure time. If your activity varies much, for example between summer and winter, then give an average. The question refers only to the last twelve months.”*  *1. Reading, watching TV, or other sedentary activity?*  *2. Walking, cycling, or other forms of exercise at least 4 hours a week? (including walking or cycling to place of school, shopping, Sunday-walking, etc.)*  *3. Participation in recreational sports, heavy outdoor activities, snow clearing etc.? (note: duration of activity at least 4 hours a week).*  *4. Participation in hard training or sports competitions, regularly several times a week?* | *0. Moderate/sports/hard training*  *1. Sedentary* |
| ***Sugar-containing sweets and beverages*** | *“How often do you usually eat sweets? (e.g. chocolate, candy)?”*  *“How often do you usually eat snacks (e.g. chips, biscuits, cakes, buns)?”*  *1. Rarely/never*  *2. 1-3 times per month*  *3. 1-3 times per week*  *4. 4-6 times per week*  *5. Every day*  *“How often do you usually drink juice with sugar? ("saft")”?*  *“How often do you usually drink soft drinks with sugar (a 1/2 liter bottle equals 2 glasses)?”*  *1. Rarely/never*  *2. 1-6 glasses per week*  *3. 1 glass per day*  *4. 2-3 glasses per day*  *5. 4 or more glasses per day* | *The scores were added and resulted in 13 groups, which were then dichotomized into:*  *0. Low sugar intake frequency (score 3-8)*  *1. High sugar intake frequency (score 9-15)* |
| ***Other dietary factors:***  ***omega 3 fatty acids-rich food/supplements,***  ***dairy products, fruits/vegetables, vitamin or mineral supplements*** | *The variable was based on the following questions, the categorized responses were added and resulted in seven categories, which further were dichotomized into unhealthy diet (score 3-5) and more healthy diet (score 6-9).* | *The final scores of the three food categories were added and resulted in 7 groups, which were then dichotomized into:*  *0. More healthy (scores 6-9)*  *1. Less healthy (scores 3-5)* |
| ***-Omega 3 fatty acids-rich food/supplements*** | *“How often do you usually eat fat fish (e.g. salmon, trout, mackerel, herring)?”*  *1. Rarely/never*  *2. 1-3 times per month*  *3. 1-3 times per week*  *4. 4-6 times per week*  *5. Every day*  *“Do you take cod liver oil, cod liver oil capsules or fish oil capsules?”(supplements)*  *1.Yes, daily*  *2. Sometimes*  *3. No* | *Answers for each question were categorized into:*  *Low frequency (no supplements/rarely or never eat fat fish or 1-3 times a month)*  *Moderate frequency (sometimes supplements/1-3 times a week fat fish)*  *High frequency (supplements daily/fat fish 4-6 times a week or daily) omega 3 fatty acids rich food/supplements intake.*  *Then the responses from both questions were combined into:*  *1.Low frequency (no supplements, AND rarely or never eat fat fish OR 1-3 times a month)*  *2. Moderate frequency (sometimes supplements AND 1-3 times a week fat fish)*  *3. High frequency (supplements daily AND fat fish 4-6 times a week or daily).* |
| ***-Dairy products*** | *“How often do you usually eat cheese (all kinds)?”*  *1. Rarely/never*  *2. 1-3 times per month*  *3. 1-3 times per week*  *4. 4-6 times per week*  *5. Every day*  *“How much do you usually drink of whole milk, kefir and yoghurt?”*  *“How often do you usually drink semi-skimmed milk, cultura and fat-reduced yoghurt?”*  *“How often do you usually drink skimmed milk (sweet or sour)?”*  *“How often do you usually drink extra semi-skimmed milk?”*  *1.Rarely/never*  *2. 1-6 glasses per week*  *3. 1 glass per day*  *4. 2-3 glasses per day*  *5. 4 or more glasses per day* | *Answers for each question were categorized into:*  *Low frequency (cheese rarely or never or 1-3 times a month/milk rarely or never)*  *Moderate frequency (cheese 1-6 times a week/milk 1-6 glasses a week)*  *High frequency (cheese every day/milk at least one glass of milk a day).*  *Then the responses were combined into:*  *1. Low frequency (cheese rarely or never or 1-3 times a month AND milk rarely or never)*  *2. Moderate frequency (cheese 1-6 times a week AND milk 1-6 glasses a week),*  *3. High frequency (cheese daily AND least one glass of milk a day).* |
| ***-Fruits, vegetables, vitamins and minerals*** | *“How often do you usually eat fruit?*  *How often do you usually eat vegetables?*  *1.Rarely/never*  *2. 1-3 times per month*  *3. 1-3 times per week*  *4. 4-6 times per week*  *5. 1-2 times per day*  *6. 3-4 times per day*  *7. 5 times or more per day*  *Do you use vitamin or mineral supplements?”*  *1.Yes, daily*  *2. Sometimes*  *3. No* | *Answers for each question were categorized into:*  *Low frequency (fruits, vegetables rarely/never or 1-3 times a month, never supplements)*  *Moderate (fruit, vegetables 1-6 times a week, sometimes supplements), High (fruits, vegetables at least 1-2 times a day, supplements daily). Then the responses were combined into:*  *1. Low frequency (never supplements AND fruits or vegetables rarely/never or 1-3 times a month)*  *2. Moderate (sometimes supplements ANd fruit or vegetables 1-6 times a week)*  *3. High (supplements daily AND fruits or vegetables at least 1-2 times a day).* |
| ***Tooth brushing frequency*** | *“How often do you usually brush your teeth?”*  *1.Less frequently than once a week*  *2. Once a week*  *3. 2 to 3 times a week*  *4. 4 to 6 times a week*  *5. Once a day*  *6. 2 or more times a day* | *0. Frequent tooth brushing (2 or more times a day)*  *1. Less frequent tooth brushing (less than 2 times a day)* |
| ***Dental satisfaction and self-esteem*** | *“Are you satisfied with the alignment of your anterior teeth?”*  *1.Very satisfied*  *2. Fairly satisfied*  *3. Neither satisfied nor dissatisfied*  *4. Fairly dissatisfied*  *4. Very dissatisfied*  *“Do you avoid smiling due to the appearance of your teeth?”*  *1.Often*  *2. Sometimes*  *3. Difficult to say*  *4. Seldom*  *5. Never*  *“I find it quite hard to make friends”*  *“I have many friends”*  *“My peers don't like me”*  *“I am popular among my peers”*  *“I feel that my peers accept me”.*  *1.Highy correct*  *2. Somewhat correct*  *3. Somewhat incorrect*  *4. Highly incorrect* | *0. High (if at least two of the following factors: very/fairly satisfied with the alignment of anterior teeth; never/seldom avoid smiling due to the appearance of your teeth; self-perceived social relation with peers is positive or somewhat positive)*  *1. Low (if less than two of the following factors: very/fairly dissatisfied/neither satisfied nor dissatisfied with the alignment of anterior teeth;*  *often/sometimes/difficult to say avoid smiling due to the appearance of your teeth; self-perceived social relation with peers is negative or somewhat negative* |
| ***Psychological therapy*** | *Have been in therapy by a psychologist, a psychiatrist or the pedagogic-psychologic services during the last year?*  *1. No*  *2. Yes* | *0.No*  *1.Yes* |
| ***Sleep sufficiency*** | *“Do you feel that you get enough sleep?”*  *1.Yes, absolutely enough*  *2. Yes, normally enough*  *3. No, somewhat insufficient*  *4. No, clearly insufficient*  *5. No, far from sufficient* | *0. Enough (absolutely enough and normally enough)*  *1. Not enough (somewhat insufficient, clearly insufficient, far from sufficient)* |
| ***Vitamin D status*** | *Measured 25-hydroxyvitamin D level in blood serum* | *0.Optimal (≥75nmol/L)*  *1. Less than optimal (<75nmol/L)* |
