# Supplementary material for: Common social determinants for overweight and obesity, and dental caries among adolescents in Northern Norway: a cross-sectional study from the Tromsø Study Fit Futures cohort
Source: BMC Oral Health. 2021 Feb 5;21:53. doi: 10.1186/s12903-021-01406-5 (PMC7866637; doi:10.1186/s12903-021-01406-5)
Supplement: Supplementary file 2 — Additional file 2: Table 2. Demographic characteristics, behavioral, biological and psychosocial factors among the participants of The Tromsø Study Fit Futures 1 (FF1) stratified by sex. [file 12903_2021_1406_MOESM2_ESM.docx]

Supplement Table 2. Demographic characteristics, behavioral, biological and psychosocial factors among the participants of The Tromsø Study Fit Futures 1 (FF1) stratified by sex.

| Characteristics | Girls | | Boys | |
| --- | --- | --- | --- | --- |
|  | FF1 |  | FF1 |  |
|  | N (%) |  | N (%) |  |
| **Demographic characteristics** |  |  |  |  |
| **Age in years^[[1]](#endnote-1)^** | 464 |  | 494 |  |
| *15* | *14 (3)* |  | *37 (8)* |  |
| *16* | *371 (80)* |  | *383 (77)* |  |
| *17* | *741(15)* |  | *58 (12)* |  |
| *18* | *8 (2)* |  | *16 (3)* |  |
| **Birth country** | 464 |  | 486 |  |
| *Norway* | *438 (95)* |  | *462 (95)* |  |
| *Other* | *24 (5)* |  | *24 (5)* |  |
| **Household composition** | 461 |  | 486 |  |
| *Without adults* | *81 (18)* |  | *74 (15)* |  |
| *With adults* | *380 (82)* |  | *412 (85)* |  |
| **Behavioral, biological and psychosocial factors** | | | |  |
| **History of chronic diseases** | 464 |  | 490 |  |
| *No* | *315 (68)* |  | *358 (73)* |  |
| *Yes* | *149 (32)* |  | *132 (27)* |  |
| **Alcohol intake*** | 462 |  | 484 |  |
| *Never* | *109 (24)* |  | *156 (32)* |  |
| *Sometimes* | *353 (76)* |  | *328 (68)* |  |
| **Smoking** | 460 |  | 486 |  |
| *Never* | *370 (80)* |  | *373 (77)* |  |
| *Sometimes/daily* | *90 (20)* |  | *113 (23)* |  |
| **Snuff use*** | 461 |  | 485 |  |
| *Never* | *310 (67)* |  | *288 (59)* |  |
| *Sometimes/daily* | *265 (57)* |  | *197 (41)* |  |
| **Physical activity*** | 462 |  | 486 |  |
| *Moderate/sports/hard training* | *400 (87)* |  | *341 (70)* |  |
| *Sedentary* | *62 (13)* |  | *145 (30)* |  |
| **Sugar-containing sweets and beverages*** | 457 |  | 480 |  |
| *Low intake frequency* | *365 (80)* |  | *320 (67)* |  |
| *High intake frequency* | *92 (20)* |  | *160 (33)* |  |
| **Other dietary factors*** | 437 |  | 465 |  |
| *More healthy* | *337 (77)* |  | *323 (70)* |  |
| *Less healthy* | *100 (23)* |  | *142 (30)* |  |
| **Tooth brushing frequency*** | 442 |  | 451 |  |
| *Frequent* | *361 (82)* |  | *280 (62)* |  |
| *Less frequent* | *81 (18)* |  | *171 (38)* |  |
| **Dental satisfaction and self-esteem** | 451 |  | 485 |  |
| *High* | *259 (57)* |  | *301 (62)* |  |
| *Low* | *192 (43)* |  | *184 (38)* |  |
| **Psychological therapy** | 461 |  | 480 |  |
| *No* | *394 (86)* |  | *449 (94)* |  |
| *Yes* | *67 (14)* |  | *31 (7)* |  |
| **Sleep sufficiency*** | 457 |  | 482 |  |
| *Enough* | *183 (40)* |  | *236 (49)* |  |
| *Not enough* | *274 (60)* |  | *246 (51)* |  |
| **Vitamin D status*** | 406 |  | 463 |  |
| *Optimal* | *77 (19)* |  | *29 (6)* |  |
| *Less than optimal* | *329 (81)* |  | *434 (94)* |  |

1. p<0.05 according to Chi-square test [↑](#endnote-ref-1)
